# The antioxidant and prebiotic properties of lactobionic acid

**DOI:** 10.1007/s00253-019-09754-7

**Published:** 2019-03-27

**Authors:** Kamila Goderska

**Affiliations:** Department of Fermentation and Biosynthesis, Institute of Food Technology of Plant Origin, Faculty of Food Science and Nutrition, Poznań University of Life Sciences, Wojska Polskiego 31, 60-624 Poznań, Poland

**Keywords:** Lactobionic acid, Prebiotic, Antioxidant activity

## Abstract

The aim of this research was to analyze the antioxidant and prebiotic properties of lactobionic acid and to develop a method of producing it from whey using the bacterium *Pseudomonas taetrolens*. Prebiotic properties were tested with selected bacterial strains that exhibit probiotic properties, while the antioxidant efficacy was tested using cold-pressed rapeseed oil. A particularly evident prebiotic effect was observed with the bacterium *Lactobacillus fermentum* with a lactobionic acid concentration of 16 mg/cm^3^. The growth curves of microorganisms in a substrate with various levels of lactobionic acid showed similarities between *Lactococcus lactis*, *Lactobacillus acidophilus* DSM 20242, *Lactobacillus acidophilus* L-AH1, *Lactobacillus acidophilus* NCDO, *Lactobacillus delbrueckii* A, *Lactobacillus casei*, *Lactobacillus casei* Shirota, *Bifidobacterium bifidum* DSM 20215, and *Bifidobacterium bifidum* DSM 20456, where a short logarithmic growth phase could be distinguished, in comparison to the growth of *Lactobacillus fermentum* and *Lactobacillus acidophilus* CH-5, where the logarithmic growth phase was extended. *Bifidobacterium bifidum* DSM 20082 and *Bifidobacterium bifidum* DSM 20239 form a separate group. The greater the amount of lactobionic acid added, the higher its activity. The greatest oxidation inhibition efficacy in rapeseed oil was recorded on day 10 of storage at 60 °C with an acid content of 10 mg/cm^3^. Expressed as a percentage reduction of peroxide value, this effect was 19.6%. The best result for preparations of lactobionic acid were found at 1 cm^3^ (22.03 mg/cm^3^), amounting to 7.3% on day 10 of the rapeseed oil thermostat test.

## Introduction

Lactobionic acid is a polyhydroxy acid composed of a molecule of galactose (a chemically neutral sugar) and a molecule of gluconic acid (naturally found in the skin and capable of retaining considerable amounts of water), containing numerous hydroxy groups. Its systematic name is 4-O-β-d-galactopyranosylo-d-gluconic acid (Gutiérrez et al. 2012). It is a weak acid with a molecular mass of 358.3 Da, a sweet taste, and an energy value of 2 kcal/g (Fox and McSweeney 2009; Schaafsma 2008). Lactobionic acid has a poorly known structure and limited applications due its high production cost. For this reason, microbiological production of this acid—which may be possible with very high efficiency and no need for enzyme purification—would seem to be an interesting alternative and a cutting-edge technique based on the latest advances in biotechnology (Satory et al. 1997). Our first study of the production of lactobionic acid used strains of *Zymomonas mobilis* and genetically engineered strains of *Escherichia coli* (Goderska et al. 2015). Due to the very limited number of reports on the production of lactobionic acid using *Pseudomonas taetrolens*, we then conducted experiments on the fermentation of whey using a strain of this microbial species (Goderska et al. 2014).

Despite being a relatively recent discovery, this technique has a variety of uses. It is important in food technology, including cheese-making and dairy industry, where it can be used to reduce the acidity of cheeses and yoghurts and to speed up cheese ripening. It is used in functional beverages as a calcium carrier and for its prebiotic properties (Belkacemi et al. 2011). It is a popular flavor enhancer. Due to its antioxidant capacity, it is used to prevent fat oxidation (Hallamaa et al. 2003). Its hygroscopic properties make it useful in the production of pudding mixes, where it can prevent caking and give better dispersion (Silva and Yang 1995). The surface-active properties of lactobionic acid (LBA) are used in the production of biodegradable detergents. An interesting application is in the production of anticorrosive agents and coverings on ships and oil platforms (Alves et al. 2011). It has a number of potential applications in medicine, pharmaceuticals, and cosmetics. Its antioxidant properties and ability to chelate metals make it useful as a component of fluids that stabilize organs intended for transplantation (Beden et al. 1995). It plays an important role in the treatment of skin diseases, such as seborrheic dermatitis, rosacea, and various types of pimples and skin lesions (Warowna et al. 2018). It is a component of preparations used after prolonged exposure to the sun, or after peeling or laser therapy. In the cosmetics industry, the moisturizing and exfoliating properties of LBA are useful, and it can help strengthen the epidermal barrier. LBA has a stimulating effect on phytoblasts, supporting the production of collagen fibrils (Warowna et al. 2018).

The aim of this study was to indicate new directions for applications of lactobionic acid. Due to the very limited number of publications on this subject, we undertook this study to investigate the production of lactobionic acid with the highest possible efficiency, while suggesting new directions for its application, providing an additional value for pure science. We thus proposed the following two research hypotheses: that lactobionic acid exhibits prebiotic properties and that lactobionic acid exhibits antioxidant properties.

Verification of the individual hypotheses included determining the characteristics of lactobionic acid as a potentially functional additive, the prebiotic properties of lactobionic acid in relation to strains of probiotic (and potentially probiotic) bacteria and the determination of the capacity of lactobionic acid to inhibit oxidation processes in rapeseed oil.

## Material and methods

### Lactobionic acid as a potentially functional addition: its prebiotic properties

The following bacterial strains exhibit probiotic or potentially probiotic properties and were used to test the prebiotic properties of lactobionic acid: *Lactococcus lactis* ATCC1 (from the Department of Fermentation and Biosynthesis, Poznań University of Life Sciences), *Lactobacillus acidophilus* DSM 20242 (Deutsche Sammlung von Microorganismen und Zellkulturen), *Lactobacillus fermentum* (Department of Fermentation and Biosynthesis, Poznań University of Life Sciences), *Lactobacillus acidophilus* CH-5 (Łódź University of Technology), *Lactobacillus acidophilus* L-AH1 (Łódź University of Technology), *Lactobacillus acidophilus* NCDO (Łódz University of Technology), *Lactobacillus delbrueckii* A (Department of Fermentation and Biosynthesis, Poznań University of Life Sciences), *Lactobacillus casei* (Department of Fermentation and Biosynthesis, Poznań University of Life Sciences), *Lactobacillus casei* Shirota (Department of Fermentation and Biosynthesis, Poznań University of Life Sciences), *Bifidobacterium bifidum* DSM 20239 (Deutsche Sammlung von Microorganismen und Zellkulturen), *Bifidobacterium bifidum* DSM 20456 (Deutsche Sammlung von Microorganismen und Zellkulturen), *Bifidobacterium bifidum* DSM 20215 (Deutsche Sammlung von Microorganismen und Zellkulturen), and *Bifidobacterium bifidum* DSM 20082 (Deutsche Sammlung von Microorganismen und Zellkulturen). Prior to analysis, the strains of probiotic bacteria were cultured in MRS broth at 37 °C for 48 h. Next, 5 cm^3^ samples were collected from the bacterial culture and were diluted in physiological saline. Selected strains at 20 μl were transferred onto microtitration plates with 150 μl medium and 30 μl lactobionic acid of varying concentrations. Analysis of the effect of lactobionic acid on the growth of selected strains of probiotic bacteria was conducted using an automatic Microplate Reader model ELx808 (Dialab, Austria). Experiments were performed in three replications for each concentration of lactobionic acid. Statistical analysis was conducted using Excel 2010 and Curve Expert Professional 2.2.0 software in order to determine an appropriate mathematical model. Mathematical models of Gompertz curves were fitted to the growth curves for probiotic bacteria in substrate with the addition of 0.1% *w*/*v* (1 mg/cm^3^), 0.2% *w*/*v* (2 mg/cm^3^), 0.5% *w*/*v* (5 mg/cm^3^), 0.8% *w*/*v* (8 mg/cm^3^), 1.0% *w*/*v* (10 mg/cm^3^), 1.2% *w*/*v* (12 mg/cm^3^), 1.4% *w*/*v* (14 mg/cm^3^), 1.6% *w*/*v* (16 mg/cm^3^), 1.8% *w*/*v* (18 mg/cm^3^), and 2.0% *w*/*v* (20 mg/cm^3^) lactobionic acid.

### Assessment of the inhibitory effects of lactobionic acid on oxidation of rapeseed oil

Prior to the study, 25 ml samples of rapeseed oil (from Kujawski ZT Kruszwica) were supplemented with lactobionic acid (Sigma-Aldrich, St. Louis, USA) dissolved in glycerol. This solution was added at various percentage levels to four samples. A lactobionic acid preparation was used in the next three samples (preparation 1, culture 3 for 96 h; preparation 2, culture 4 for 96 h; preparation 3, culture 7 for 24 h) on 1 cm^3^. Next, the samples were incubated at a temperature of 60 °C in accordance with the Schaal thermostat test. Peroxide values were determined in these samples after 1 day, 3 days, 5 days, and 10 days. The assay was performed in accordance with the appropriate Polish standard (PN-EN ISO 3960:2012). The peroxide value is the number of millimoles of active oxygen contained in 1.0 g oil. It is expressed as the Lea value, which is determined by the volume in cubic centimeters of sodium thiosulfate solution (0.002 mol/dm^3^) used in the titration of iodine released from potassium iodide as a result of the action of the peroxides contained in 1.0 g fat (Pomeranz and Meloan 1994). Statistical analysis was conducted using Excel 2010 software.

## Results

In analyzing the growth curves for the tested microorganisms in the substrate with various levels of lactobionic acid, we found similarities between *Lactococcus lactis*, *Lactobacillus acidophilus* DSM 20242, *Lactobacillus acidophilus* L-AH1, *Lactobacillus acidophilus* NCDO, *Lactobacillus delbrueckii* A, *Lactobacillus casei*, *Lactobacillus casei* Shirota, *Bifidobacterium bifidum* DSM 20215, and *Bifidobacterium bifidum* DSM 20456, where a short logarithmic growth phase was distinguished, unlike in *Lactobacillus fermentum* and *Lactobacillus acidophilus* CH-5, where the logarithmic growth phase was extended. *Bifidobacterium bifidum* DSM 20082 and *Bifidobacterium bifidum* DSM 20239 made up a separate group, in which an extension of the logarithmic growth phase was observed upon the addition of 0.5% *w*/*v* (5 mg/cm^3^), 1.0% *w*/*v* (10 mg/cm^3^), and 1.2% *w*/*v* (12 mg/cm^3^) lactobionic acid for strain 1 and 1.0% *w*/*v* (10 mg/cm^3^) and 1.4% *w*/*v* (14 mg/cm^3^) lactobionic acid for strain 2. The logarithmic growth phase was much shorter in the medium with the addition of other amounts of lactobionic acid.

Our research confirms that lactobionic acid has the capacity to stimulate the growth of probiotic bacteria and may be considered as a prebiotic substance (Saarela et al. 2003). The varied quantitative addition of lactobionic acid had a decisive effect on growth dynamics of the tested probiotic bacteria; growth in a medium containing LBA depends on the microbial strain. The results suggest that lactobionic acid might serve as a new prebiotic.

**Fig. 1:**
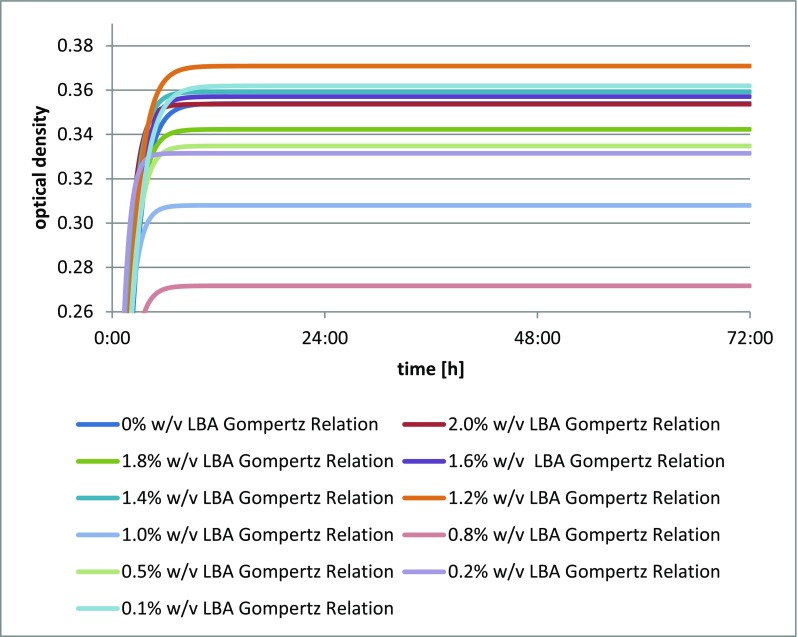
Changes in optical density in *Lactococcus lactis* ATCC1 culture depending on the amount of lactobionic acid added. A 0.1% addition (*w*/*v*) is equivalent to 1 mg/cm^3^ lactobionic acid

The strain *Lactococcus lactis* showed a slight increase in biomass when lactobionic acid was added, compared to when it was not. The use of a substrate with the addition of 1.2% (*w*/*v*; 12 mg/cm^3^) of LBA showed the greatest optical density of this culture, while the others led to densities almost equal to or lower than that of the blank test (Fig. 1).

**Fig. 2:**
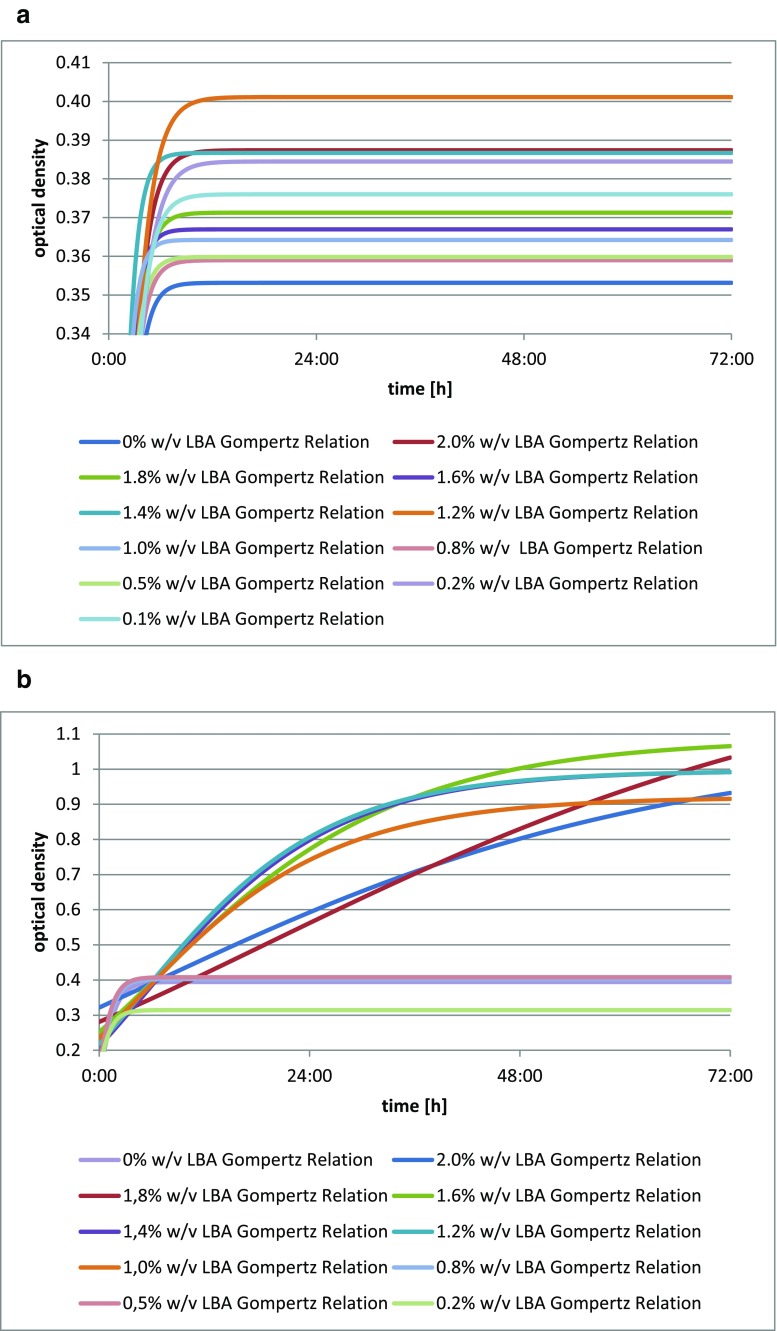

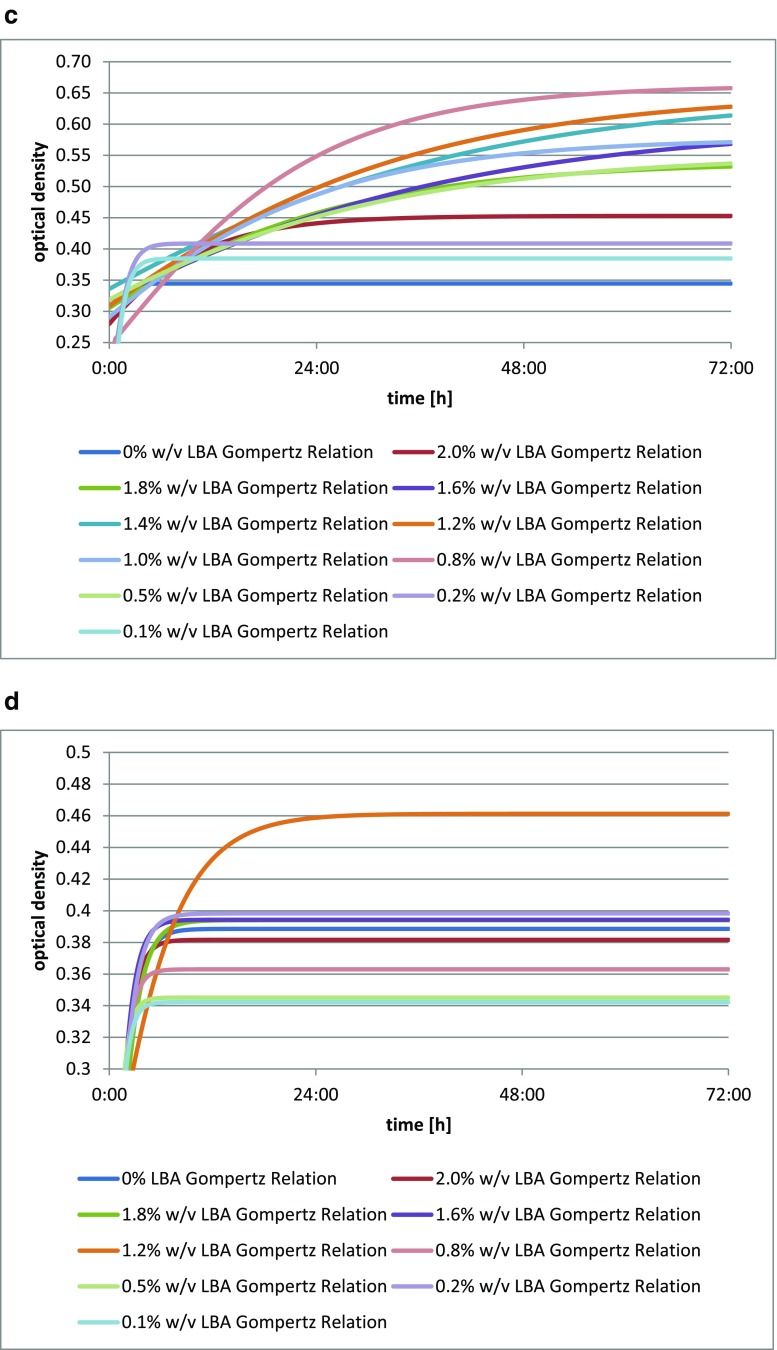

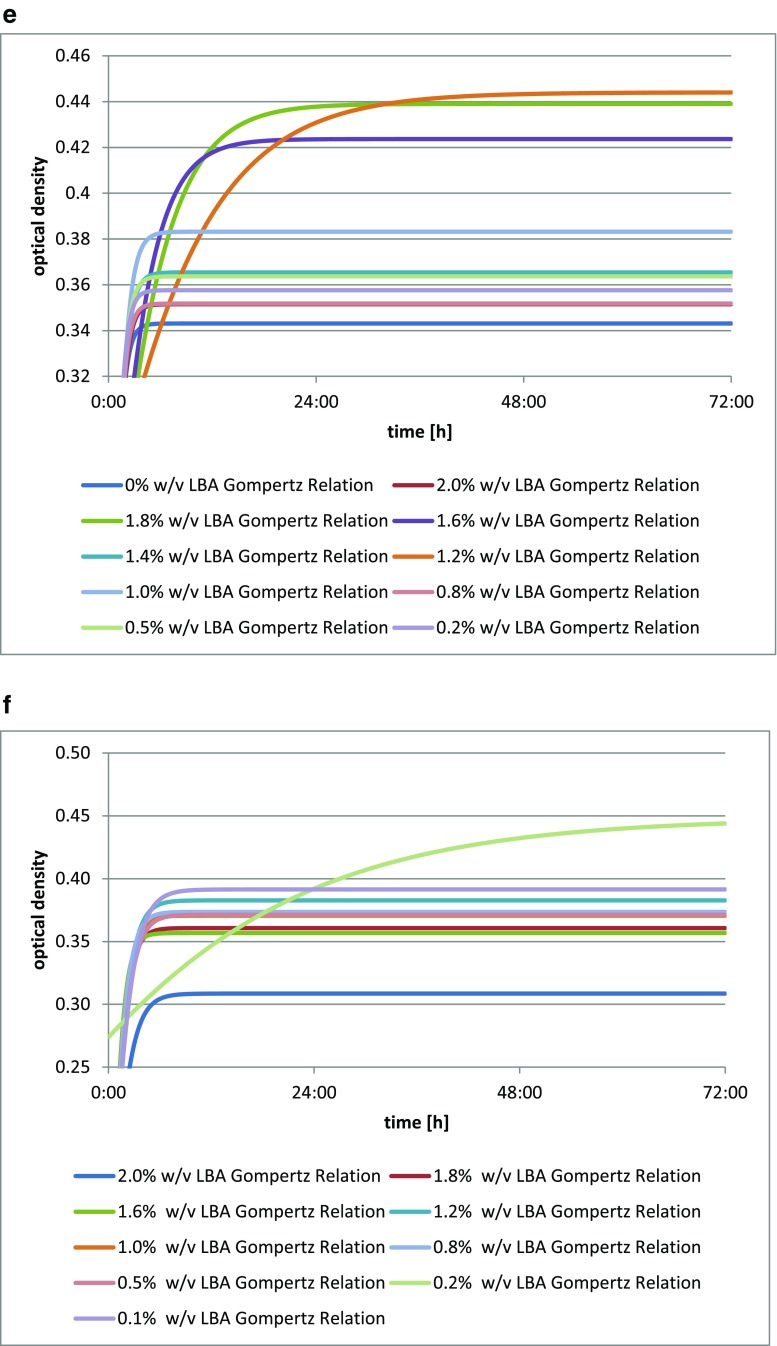

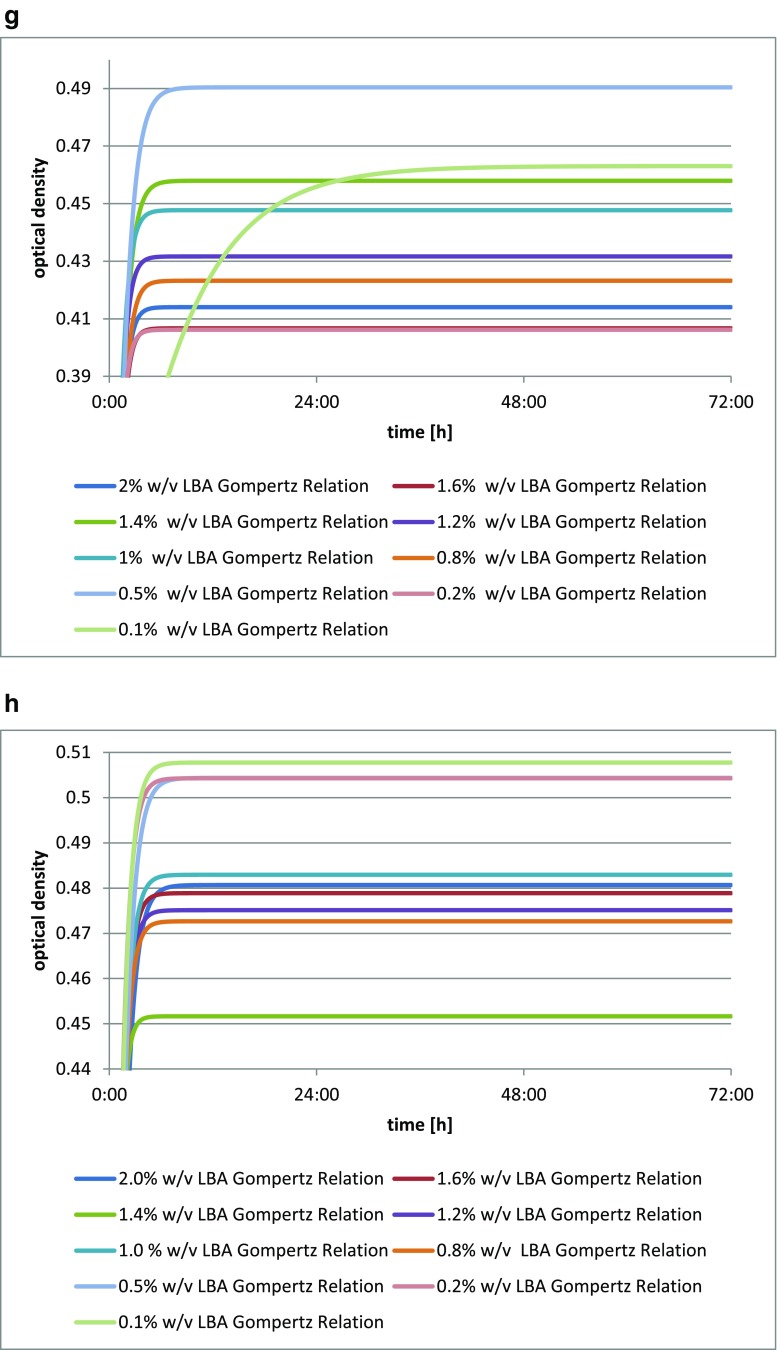
**a** Changes in optical density in *Lactobacillus acidophilus* DSM 20242 culture depending on the amount of lactobionic acid added. A 0.1% addition (*w*/*v*) is equivalent to 1 mg/cm^3^ lactobionic acid. **b** Changes in optical density in *Lactobacillus fermentum* culture depending on the amount of lactobionic acid added. A 0.1% addition (*w*/*v*) is equivalent to 1 mg/cm^3^ lactobionic acid. **c** Changes in optical density in *Lactobacillus acidophilus* CH-5 culture depending on the amount of lactobionic acid added. A 0.1% addition (*w*/*v*) is equivalent to 1 mg/cm^3^ lactobionic acid. **d** Changes in optical density in *Lactobacillus acidophilus* L-AH1 culture depending on the amount of lactobionic acid added. A 0.1% addition (*w*/*v*) is equivalent to 1 mg/cm^3^ lactobionic acid. **e** Changes in optical density in *Lactobacillus acidophilus* NCDO culture depending on the amount of lactobionic acid added. A 0.1% addition (*w*/*v*) is equivalent to 1 mg/cm^3^ lactobionic acid. **f** Changes in optical density in *Lactobacillus delbrueckii* A culture depending on the amount of lactobionic acid added. A 0.1% addition (*w*/*v*) is equivalent to 1 mg/cm^3^ lactobionic acid. **g** Changes in optical density in *Lactobacillus casei* culture depending on the amount of lactobionic acid added. A 0.1% addition (*w*/*v*) is equivalent to 1 mg/cm^3^ lactobionic acid. **h** Changes in optical density in *Lactobacillus casei* Shirota culture depending on the amount of lactobionic acid added. A 0.1% addition (*w*/*v*) is equivalent to 1 mg/cm^3^ lactobionic acid

The addition of lactobionic acid stimulated the growth of *Lactobacillus acidophilus* DSM 20242, as shown by the increase in biomass in each culture with varying levels of the acid. A reference for the comparison was provided by the blank test. The greatest optical density was found for *Lactobacillus acidophilus* DSM 20242 with the addition of 1.2% *w*/*v* (12 mg/cm^3^) of LBA (Fig. 2a).

The addition of lactobionic acid stimulated the growth of *Lactobacillus fermentum* culture, as indicated by changes in the optical density of the medium for the culture in comparison to the blank test (Fig. 2b). The greatest and also the latest increase in biomass after 72 h was found for *Lactobacillus fermentum* with the addition of 1.6% *w*/*v* (16 mg/cm^3^) of LBA. This strain had the lowest increase in optical density when cultured on the substrate with the lowest LBA addition.

The strain *Lactobacillus acidophilus* exhibited an increase increment in biomass when LBA was added, as was clearly evidenced by the growth curve for each culture with varying levels of the acid (Fig. 2c). The growth curve for the blank test was used for comparison. The optical density of the culture of *Lactobacillus acidophilus* CH-5 was stimulated to the greatest extent by the addition of 0.8% *w*/*v* (8 mg/cm^3^) of LBA. Additionally, this strain reached its latest maximum increase in biomass within the 72-h period.

The addition of lactobionic acid, to a limited extent, stimulates growth in the culture of *Lactobacillus acidophilus* L-AH1 (Fig. 2d). This is indicated by changes in optical density of the medium in which cultures were run. A reference was provided by the test with no acid added. One exception was observed for *Lactobacillus acidophilus* L-AH1 with the addition of 1.2% *w*/*v* (12 mg/cm^3^) of LBA, which exhibited the greatest and also the latest increase in biomass. Growth curves in other media were almost equal to or lower than the curves of the blank test.

The strain *Lactobacillus acidophilus* NCDO exhibited an increase in biomass when LBA is used (Fig. 2e), as confirmed by the growth curve for each culture with varying levels of the acid in comparison to the blank test. The optical density of the *Lactobacillus acidophilus* NCDO culture was most strongly (and also the latest) stimulated by the addition of 1.2% *w*/*v* (12 mg/cm^3^) LBA, while this was lowest at the 0.8% *w*/*v* level.

The strain *Lactobacillus delbrueckii* A showed an increase in biomass with the addition of LBA (Fig. 2f), as shown by changes in the optical density of the medium in which the cultures were run. The most optimal growth curve and also the highest latest optical density were seen for *Lactobacillus delbrueckii* A with the addition of 0.2% *w*/*v* (2 mg/cm^3^) LBA. On media with the highest levels LBA added, this strain had the lowest growth curve, which may mean that small amounts of lactobionic acid provide more advantageous growth conditions for this strain.

The addition of lactobionic acid stimulated the growth of the *Lactobacillus casei* culture (Fig. 2g), as was clearly evidenced by the growth curve for each culture with the varying LBA additions. The greatest increase in biomass after 72 h was seen for *Lactobacillus casei* in the medium with the addition of 0.5% *w*/*v* (5 mg/cm^3^) of the acid, while this strain in the medium with the lowest level of acid reached its latest maximum activity level.

The strain *Lactobacillus casei* Shirota showed an increase in biomass when LBA was added (Fig. 2h), as evidenced by changes in optical density of the culture. The greatest optical density of this culture was recorded for the addition of 0.1% *w*/*v* (1 mg/cm^3^) of the acid, while this was lowest for the addition of 1.4% *w*/*v* (14 mg/cm^3^). Those strains with the greatest LBA additions were characterized by the lowest growth curves. This may mean that small amounts of lactobionic acid provide more advantageous growth conditions for these strains.

**Fig. 3:**
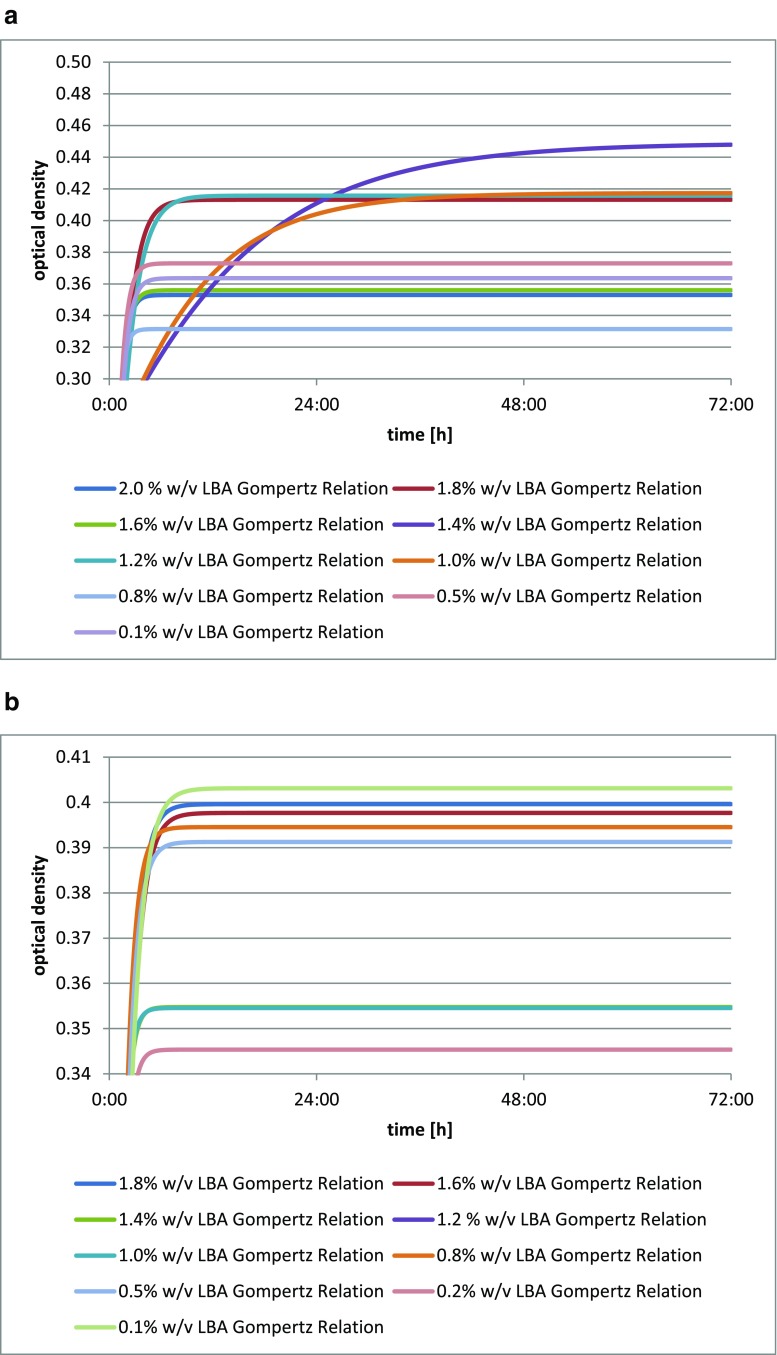

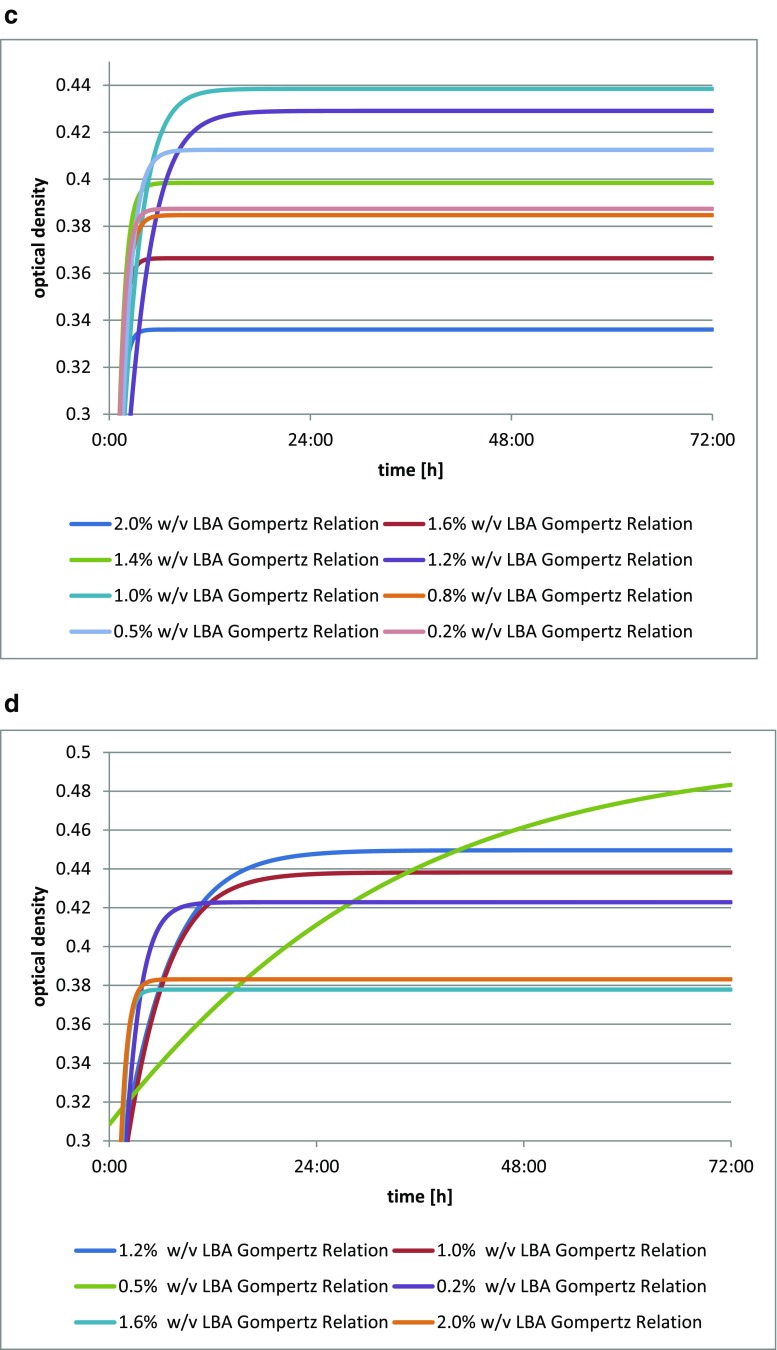
**a** Changes in optical density in *Bifidobacterium bifidum* DSM 20239 culture depending on the amount of lactobionic acid added. A 0.1% addition (*w*/*v*) is equivalent to 1 mg/cm^3^ lactobionic acid. **b** Changes in optical density in *Bifidobacterium bifidum* DSM 20456 culture depending on the amount of lactobionic acid added. A 0.1% addition (*w*/*v*) is equivalent to 1 mg/cm^3^ lactobionic acid. **c** Changes in optical density in *Bifidobacterium bifidum* DSM 20215 culture depending on the amount of lactobionic acid added. A 0.1% addition (*w*/*v*) is equivalent to 1 mg/cm^3^ lactobionic acid. **d** Changes in optical density in *Bifidobacterium bifidum* DSM 20082 culture depending on the amount of lactobionic acid added. A 0.1% addition (*w*/*v*) is equivalent to 1 mg/cm^3^ lactobionic acid

The addition of lactobionic acid stimulated the growth of the *Bifidobacterium bifidum* DSM 20239 culture (Fig. 3a), as shown by changes in optical density of the culture medium. The greatest increment in the biomass after 72 h was observed for *Bifidobacterium bifidum* DSM 20239 with the addition of 1.4% *w*/*v* (14 mg/cm^3^) of LBA. The latest maximum increase in the optical density of the culture was obtained for the media with the addition of both 1.4% *w*/*v* (14 mg/cm^3^) and 1.0% *w*/*v* (10 mg/cm^3^) of LBA.

The addition of lactobionic acid stimulated the growth of *Bifidobacterium bifidum* DSM 20456 culture (Fig. 3b), as shown by changes in the optical density of the liquid culture media. The greatest increment in biomass was seen for *Bifidobacterium bifidum* DSM 20456 with the addition of 0.1% *w*/*v* LBA (1 mg/cm^3^), while this was lowest for a 0.2% addition *w*/*v* (2 mg/cm^3^).

The strain *Bifidobacterium bifidum* DSM 20215 showed an increase in biomass with the addition of LBA (Fig. 3c), as evidenced by changes in the optical density of liquid culture media. The highest growth curve was found for *Bifidobacterium bifidum* DSM 20215 in the medium with the addition of 1.0% *w*/*v* of the acid (10 mg/cm^3^), while this was lowest with the addition of 2.0% *w*/*v* (20 mg/cm^3^) of LBA.

The *Bifidobacterium bifidum* DSM 20082 strain shows an increase in biomass (Fig. 3d) with the addition of LBA, as clearly shown by the growth curves for each culture with varying additions of the acid. The greatest optical density of the culture after 72 h, as well as the latest increase, were obtained for *Bifidobacterium bifidum* DSM 20082 with the acid added at 0.5% *w*/*v* (5 mg/cm^3^); this was lowest in the medium with the addition of 1.6% *w*/*v* of lactobionic acid (16 mg/cm^3^).

Our results confirm that prebiotic properties are strain-dependent, which means that they can be rationally utilized only for a known action of a specific probiotic strain. The decisive factors are dose, prebiotic species, and probiotic species, and so each strain needs to be treated individually. The correct selection of a prebiotic so as to stimulate the growth of the probiotic bacteria to the greatest possible extent can facilitate the development of a synbiotic.

**Fig. 4:**
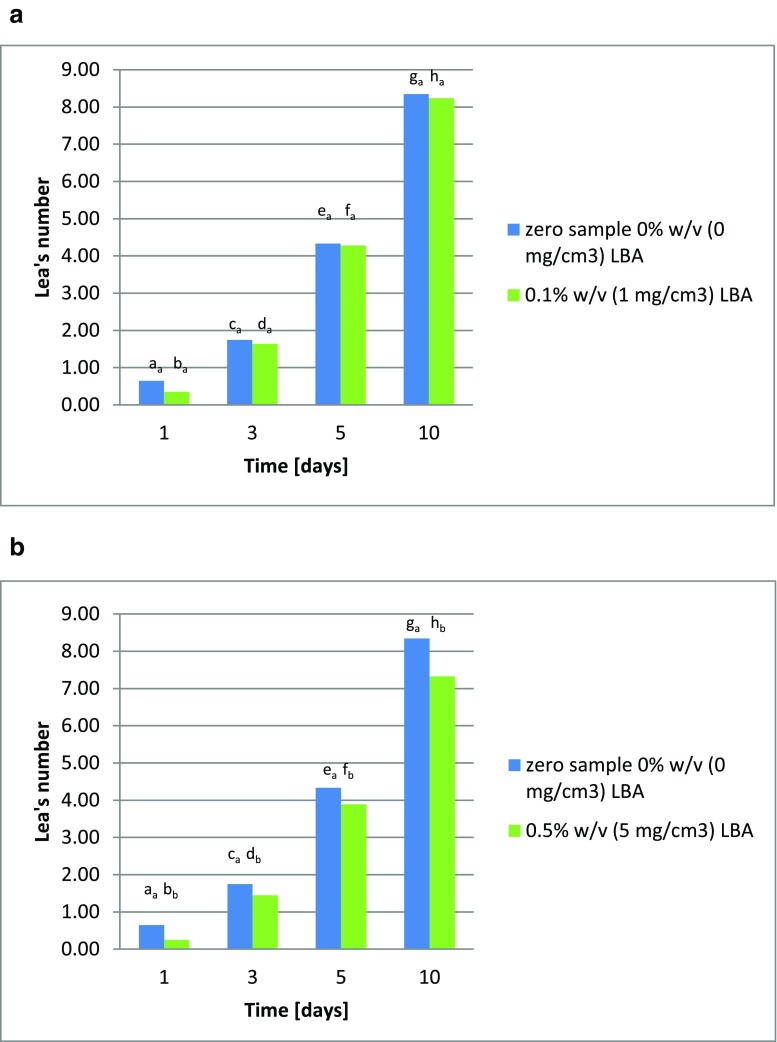

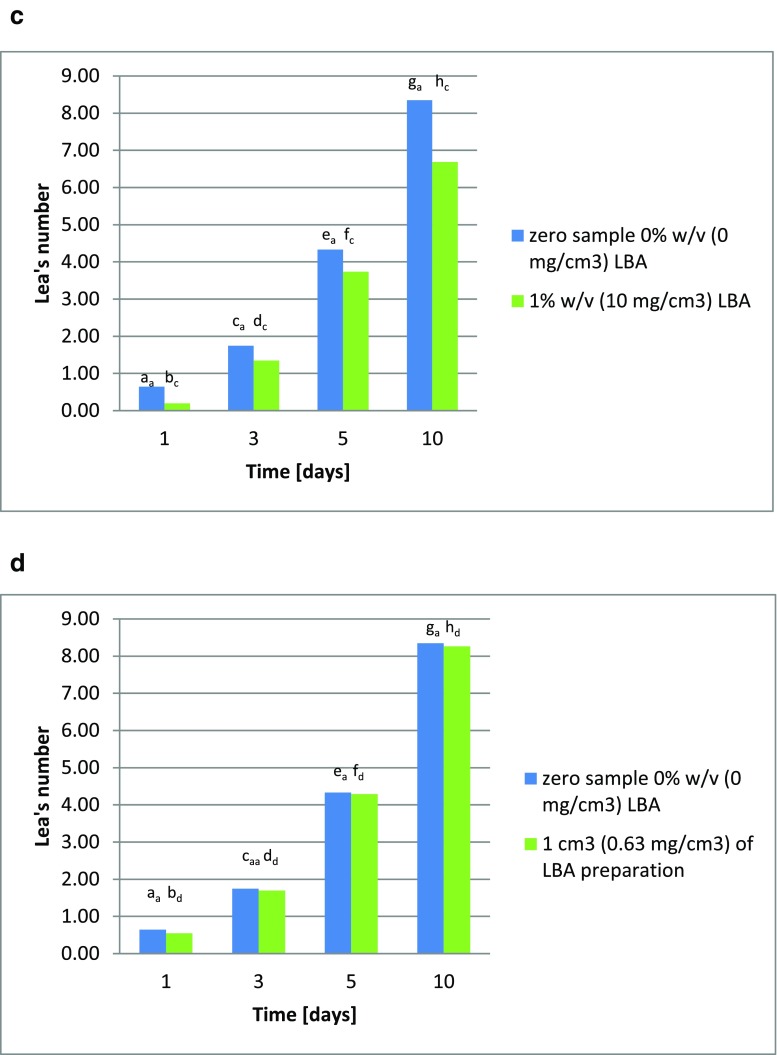

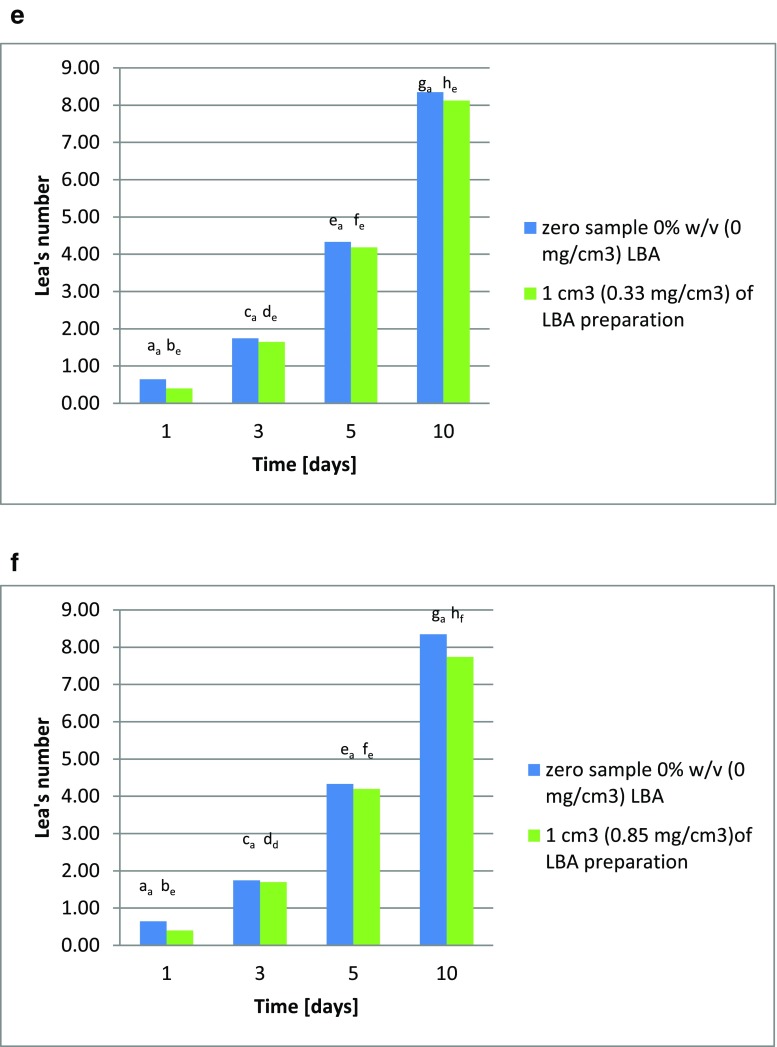
**a** Changes in peroxide value in rapeseed oil supplemented with 0.1% lactobionic acid *w*/*v* (1 mg/cm^3^). The indices a_a_–h_a_ denote significant differences between peroxide values in rapeseed oil. **b** Changes in peroxide value in rapeseed oil supplemented with 0.5% lactobionic acid *w*/*v* (5 mg/cm^3^). The indices a_a_–h_b_ denote significant differences between peroxide values in rapeseed oil. **c** Changes in peroxide value in rapeseed oil supplemented with 1% lactobionic acid *w*/*v* (10 mg/cm^3^). The indices a_a_–h_c_ denote significant differences between peroxide values in rapeseed oil. **d** Changes in peroxide value in rapeseed oil supplemented with the original preparation of lactobionic acid (collected at hour 96 of culture 3) at a concentration of 0.63 mg/cm^3^, in comparison to the blank test. The indices a_a_–h_d_ denote significant differences between peroxide values in rapeseed oil. **e** Changes in peroxide value in rapeseed oil supplemented with the original preparation of lactobionic acid (collected at hour 96 of culture 4) at a concentration of 0.33 mg/cm^3^, in comparison to the blank test. The indices a_a_–h_e_ denote significant differences between peroxide values in rapeseed oil. **f** Changes in peroxide value in rapeseed oil supplemented with the original preparation of lactobionic acid (collected at hour 24 of culture 7) at a concentration of 0.85 mg/cm^3^, in comparison to the blank test. The indices a_a_–h_f_ denote significant differences between peroxide values in rapeseed oil

The addition of lactobionic acid inhibits oxidation processes in rapeseed oil, as has also been described by Hallamaa et al. (2003). This was evidenced by a reduction in peroxide value in samples of this oil supplemented with the acid, in comparison to a sample without LBA addition (the blank test). The higher the concentration of lactobionic acid, the greater its reduction of the oxidation rate. Samples of oil with the addition of 1 cm^3^ of one of the lactobionic acid preparations (preparation 1: culture 3 for 96 h; preparation 2: culture 4 for 96 h; or preparation 3: culture 7 for 24 h) were less effective in reducing of peroxide value than samples with varying amounts of this acid. During storage of rapeseed oil at 60 °C, the Lea value increases, after ten days reaching 8.35. The addition of lactobionic acid at 0.1% *w*/*v* (1 mg/cm^3^) reduced the Lea value during storage at 60 °C, so that it reached 8.24 on day 10 (Fig. 4a). Increasing the percentage of lactobionic acid in the rapeseed oil increased the inhibition of the oxidation, with the Lea value on day 10 of 60 °C storage reaching 7.33, with the addition of 0.5 % *w*/*v* LBA (5 mg/cm^3^; Fig. 4b). The highest level of lactobionic acid (1% *w*/*v* or 10 mg/cm^3^) led to a reduction in the Lea value on day 10 to 6.69 (Fig. 4c). During the experiments, it was also decided to test the lactobionic acid preparations produced by microbial synthesis in both processes; here *Pseudomonas taetrolens* bacteria were used both in the free and encapsulated form. All the preparations reduced the Lea value for each day of oil storage at 60 °C, as compared to the samples to which no lactobionic acid preparation was added. On day 10, the Lea value ranged from 8.26 to 7.74 (Fig. 4d–f).

These changes in the peroxide value of rapeseed oil upon the addition of lactobionic acid confirm its antioxidant properties, and this relationship may find application in the fats industry to prevent oxidation of lipids. The 60 °C thermostat test provides results similar to those from storing oil for several weeks or even months. Such durations are impractical, so fats are typically tested using accelerated aging tests at higher temperatures (Trojáková et al. 2001). For this reason, a decrease of 12.2% in the peroxide value upon the addition of 0.5% lactobionic acid *w*/*v* (5 mg/cm^3^) to oil and a decrease of 19.9% upon the addition of 1% *w*/*v* (10 mg/cm^3^) of the acid on day 10 of the Schaal test seem satisfactory, assuming they are correctly replicate storage for several months under normal conditions. The best results in this study for lactobionic acid preparations were found for preparation 3 added at 1 cm^3^ (22.03 mg/cm^3^), which yielded a result of 7.3% on day 10 of the rapeseed oil thermostat test.

## Discussion

The study showed that LBA was capable of stimulating the growth of probiotic bacteria. We demonstrated that this additive significantly affected the biomass yield and bacterial growth dynamics, whereas the growth kinetics depended on the strain of microorganisms. The research confirmed that lactobionic acid stimulated the development of selected strains of probiotic and potentially probiotic bacteria (*Bifidobacterium* and *Lactobacillus*). *Lactobacillus fermentum* growing on a medium containing 16 mg/cm^3^ of lactobionic acid had the greatest increase in biomass. Our research findings confirmed that LBA stimulates the growth of bacteria of the *Bifidobacterium* genus, as suggested by published by Horton (1995) and Nakano et al. (2006).

Interest continues to increase in prebiotics, not only because they stimulate the growth of probiotic bacteria but also because they possess interesting technical properties, such as gelling, loosening, texturing, and emulsification (Śliżewska et al. 2013). For this reason, they are often used to improve the consistency of food products. They are added to a wide range of products, such as sweeteners, chewing gums, yoghurts, breads, chocolates, dietetic products, and food mixes for children. Prebiotics can be added to cakes and breads to improve their consistency and shelf-life and to reduce their caloric value. They are used in yoghurts and ice cream to improve their taste and stability. Prebiotics affect the juiciness and fat content of cold cuts and sausages (Walter 1999). As overweight and obesity are common problems at present, an increasing number of people are paying attention to the composition of products, especially to their caloric value. One of the most important features of prebiotics is their ability to replace fat and sugar without affecting the sensory qualities of finished products. Prebiotics have various important functional features that are useful in food technology. However, their prebiotic properties are strain-dependent, so they can only reasonably be used when their effect on a specific probiotic strain is known. The effect depends on the dose, the type of prebiotic, and the species of probiotic microorganism; each strain needs to be tested individually. Correct choice of a prebiotic to optimally stimulate the growth of a given probiotic bacteria allows this combination to be used as a symbiotic, and indeed, there are an increasing number of synbiotics available on the market. Prebiotics do not only stimulate the growth of probiotic bacteria but also affect their survival in products during storage. The prospect of using lactobionic acid as a prebiotic seems to be promising.
